# Circulating plasma galectin-3 predicts new-onset atrial fibrillation in patients after acute myocardial infarction during hospitalization

**DOI:** 10.1186/s12872-022-02827-y

**Published:** 2022-09-03

**Authors:** Qianhui Wang, Wei Huai, Xiaoguang Ye, Yuxia Pan, Xinchun Yang, Mulei Chen, Qing-Bian Ma, Yuanfeng Gao, Yuan Zhang

**Affiliations:** 1 Heart Center and Beijing Key Laboratory of Hypertension, Department of Cardiology, Chaoyang Hospital, Capital Medical University, 8th Gongtinanlu Rd, Chaoyang District, Beijing, 100020 China; 2 Emergency Department, Third Clinical Medical College, Peking University, Beijing, 100191 China

**Keywords:** Galectin-3, Acute myocardial infarction, New-onset atrial fibrillation, Biomarker

## Abstract

**Background:**

New-onset atrial fibrillation (NOAF) is a common complication in patients with acute myocardial infarction (AMI) during hospitalization. Galectin-3 (Gal-3) is a novel inflammation marker that is significantly associated with AF. The association between post-AMI NOAF and Gal-3 during hospitalization is yet unclear.

**Objective:**

The present study aimed to investigate the predictive value of plasma Gal-3 for post-AMI NOAF.

**Methods:**

A total of 217 consecutive patients admitted with AMI were included in this retrospective study. Peripheral venous blood samples were obtained within 24 h after admission and plasma Gal-3 concentrations were measured.

**Results:**

Post-AMI NOAF occurred in 18 patients in this study. Patients with NOAF were older (*p* < 0.001) than those without. A higher level of the peak brain natriuretic peptide (BNP) (*p* < 0.001) and Gal-3 (*p* < 0.001) and a lower low-density lipoprotein cholesterol level (LDL-C) (*p* = 0.030), and an estimated glomerular filtration rate (e-GFR) (*p* = 0.030) were recorded in patients with post-AMI NOAF. Echocardiographic information revealed that patients with NOAF had a significantly decreased left ventricular eject fraction (LVEF) (*p* < 0.001) and an increased left atrial diameter (LAD) (*p* = 0.004) than those without NOAF. The receiver operating characteristic (ROC) curve analysis revealed a significantly higher value of plasma Gal-3 in the diagnosis of NOAF for patients with AMI during hospitalization (area under the curve (*p* < 0.001), with a sensitivity of 72.22% and a specificity of 72.22%, respectively. Multivariate logistic regression model analysis indicated that age (*p* = 0.045), plasma Gal-3 (*p* = 0.018), and LAD (*p* = 0.014) were independent predictors of post-MI NOAF.

**Conclusions:**

Plasma Gal-3 concentration is an independent predictor of post-MI NOAF.

## Background

New-onset atrial fibrillation (NOAF) is the most common tachycardiac arrhythmia in patients with acute myocardial infarction (AMI) [1]. Previous studies reported that NOAF occurred in 4–20% of patients with AMI who were free from AF prior to hospitalization, and NOAF was significantly associated with poor clinical outcomes, including all-cause death, heart failure (HF), and increased risk of ischemic stroke [2, 3]. Therefore, an effective risk stratification tool for NOAF is essential for patients with AMI.

Cardiac remodeling, including structural and electrical, has been recognized as the key mechanism in the incidence and maintenance of AF [4, 5]. Atrial fibrosis plays a key role in initiating structural remodeling of the atrium and, in turn, promoting electrical remodeling. Galectin-3 (Gal-3) is a lectin binding with β-galactoside that is secreted mainly by activated macrophages and fibroblasts. Some studies reported that elevated Gal-3 concentration was significantly involved in the regulation of several fibrosis conditions, including myocardial fibrosis [6, 7]. Circulating Gal-3 concentration has been widely recognized as the biomarker of fibrotic protein.

Previous studies have shown that elevated circulating Gal-3 was significantly associated with the incidence, progress and recurrence of AF after catheter ablation [8, 9]. On the other hand, recent studies also reported that AMI patients with AF had a significantly higher level of circulating Gal-3 concentration, and Gal-3 is associated with cardiac fibrosis after myocardial infarction [10, 11]. However, the association between post-MI AF and plasma Gal-3 seems to be reasonable but has not yet been investigated. The present study aimed to compare the characteristics of AIM patients with and without NOAF during hospitalization and investigate the association between plasma Gal-3 concentration and NOAF in patients with AMI.

## Methods

### Study population

In this study, a total of 221 patients (May–November 2019) were admitted with AMI (142 ST-segment elevation myocardial infarction (STEMI) and 79 non-ST-segment elevation myocardial infarction (NSTEMI), respectively) and did not have AF previously. All subjects underwent percutaneous coronary intervention therapy (PCI) in this study. AMI and NOAF were diagnosed according to the current clinical guidelines of ESC [12]. Then, continuous electrocardiographic monitoring was provided to all patients to detect and record any AF during hospitalization. This study was approved by the local Ethics Committees of the Beijing Chaoyang Hospital, Capital Medical University, China. Written consent was obtained from all subjects included in this study.

Patients who met the following conditions were excluded from this study: pre-existing AF or presented AF at admission, acute or chronic inflammation conditions, valvular heart disease, thyroid dysfunction, malignant tumors and chronic HF. Peripheral venous blood samples were taken within 24 h after admission. Plasma was separated from whole blood by centrifugation at 4° C and 3000 rpm for 10 min and stored at -80° C for subsequent analysis.

AF was defined according to the current guidelines for the management of AF: absence of wave P with irregular RR interval lasting for at least > 30 s recorded by ECG or electrocardiographic monitoring. NOAF was defined as patients with no pre-existing history of AF and who developed AF during hospitalization.

### Gal3 measurement

The plasma level of Gal-3 was measured using commercially available enzyme-linked immunosorbent assay (ELISA) kits (Immunoway (USA) KE1712) according to the manufacturer’s instructions. Furthermore, the maximum level of c-TnI and BNP presented the degree of damage to cardiac function. Echocardiography parameters, including left ventricular end-systolic diameter (LVESD), left ventricular end-diastolic diameter (LVEDD), left atrial diameter (LAD), and left ventricular ejection fraction (LVEF), were measured by transthoracic echocardiography (GE Healthcare Life Sciences, Connecticut, USA) within 3 days after admission.

### Statistical methods

Continuous variables were presented as means ± standard deviation (SD) or medians (interquartile range) according to the normality distribution of the variable. The normality distribution of continuous variables was tested using the Shapiro–Wilk method. Categorical variables were shown as numbers (%). The statistical differences between groups were evaluated using the t test or Mann–Whitney *U* test for continuous variables and Pearson’s χ^2^ test for categorical variables. A step-by-step multivariate logistic regression model was used to determine the independent risk factors for NOAF after AMI. The receiver operating characteristic (ROC) curve analysis assessed the Gal-3 discrimination ability. SPSS v22.0 (SPSS Inc., Chicago, IL, USA) was used for statistical calculations and illustrations. All tests were two-sided, and *p* < 0.05 was considered statistically significant.

## Results

### Baseline characteristics of the subjects

In the present study, 4 patients were excluded from the cohort due to a history of AF, and a total of 217 patients were enrolled for the final analysis. In this study, the median time from AMI to the occurrence of NOAF was 3 days. The comparison of baseline clinical characteristics between patients with and without NOAF is summarized in Table 1. Post-AMI NOAF occurred in 18 (8.3%) patients during the index hospitalization. The mean age of the study cohort was 58.2 years and 33 (15.2%) patients were women. During hospitalization, NOAF patients were older (57.5 ± 10.8 vs. 66.8 ± 10.0, *p* < 0.001), had a high degree of initial Killip class, and a lower level of systolic blood pressure (117.6 ± 18.9 vs. 127.8 ± 19.7, *p* = 0.035) compared to those admitted without NOAF. On laboratory examination, a high level of BNP (188.0 vs. 865.0, *p* < 0.001) and Gal-3 (16.6 ± 6.0 vs. 22.3 ± 5.1, *p* < 0.001) and a low level of low-density lipoprotein cholesterol (LDL-C) (3.0 ± 1.0 vs. 2.5 ± 1.0, *p* = 0.030) and an estimated glomerular filtration rate (e-GFR) (97.2 ± 19.4 vs. 79.5 ± 31.1, *p* = 0.030) was observed in patients who developed NOAF. Echocardiographic information revealed that NOAF patients had a significantly decreased LVEF (59.2 ± 10.9 ± 47.7 ± 11.9, *p* < 0.001) and enlarged LAD (35.3 ± 3.8 vs. 39.2 ± 3.9, *p* = 0.004), LVESD (32.2 ± 5.6 vs. 36.4 ± 6.1, *p* = 0.003), and LVEDD (47.8 ± 4.2 vs. 50.7 ± 4.4, *p* = 0.006) than those without NOAF. Furthermore, patients with NOAF received less beta-blockers (β-blockers) (68.8% vs. 38.8%, *p* = 0.010) and ACEI/ARB (39.7% vs 11.1%, *p* = 0.016) treatment during hospitalization. Furthermore, we also observed a significant difference in the Gal-3 level in patients with STEMI and NSTEMI (18.3 ± 6.1 vs. 14.8 ± 5.7, *p* < 0.001).

**Table 1:** Baseline characteristics of patients with and without post-MI NAOF

Variables	Sinus rhythm (n = 199)	NOAF (n = 18)	*p* value
Age(years)	57.5 ± 10.8	66.8 ± 10.0	< 0.001
Male (%)	170 (85.4%)	14 (77.8%)	0.387
Hypertension (%)	104 (52.3%)	10 (55.6%)	0.789
Diabetes (%)	66 (33.2%)	7 (38.9%)	0.623
Previous Stroke/TIA (%)	25 (12.6%)	2 (11.1%)	0.858
Previous MI (%)	31 (15.6%)	5 (27.8%)	0.183
*Killip class *(*%*)			0.008
I	103 (51.8%)	9 (50.0%)	
II	87 (43.7%)	5 (27.8%)	
III–IV	9 (4.5%)	4 (22.2%)	
STEMI (%)	125 (62.8%)	13 (72.2%)	0.427
SBP (mmHg)	127.8 ± 19.7	117.6 ± 18.9	0.035
HR (bpm)	75.4 ± 13.1	77.7 ± 12.1	0.449

NOAF, new-onset atrial fibrillation; TIA, transient ischemic attacks; MI, myocardial infarction; STEMI, ST-elevation myocardial infarction; SBP, systolic blood pressure; HR, heart rate

**Fig. 1:**
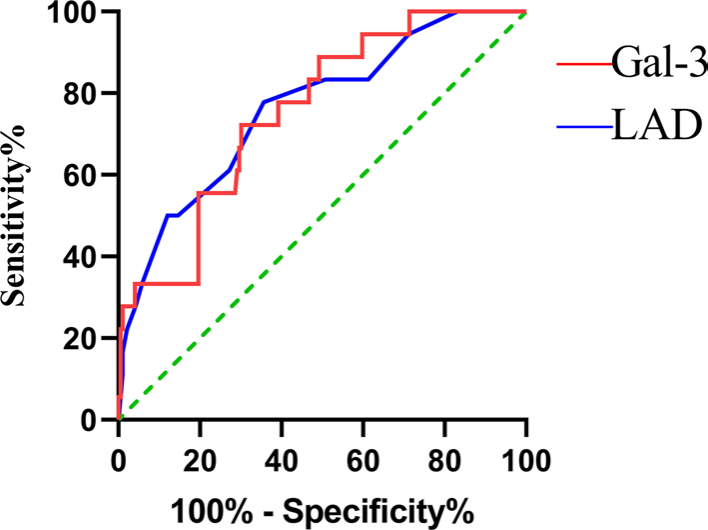
ROC curve analysis showed significant predictive value of Gal-3 and LAD for the incidence of post-AMI NOAF

The ROC curve analysis indicated that both plasma Gal-3 (area under curve AUC) (C index) = 0.756, 95% confidence interval (CI):0.650–0.861, *p* < 0.001) and LAD (AUC = 0.763, 95% CI 0.645–0.880, *p* < 0.001) were strong independent predictors of NOAF diagnosis after MI during hospitalization (Fig. 1). The optimal cut-off values for Gal-3 and LAD were 20.10 ng/ml and 36.5 mm, with sensitivity and specificity 72.22%, 78.89% and 72.22%, 60.73%, respectively.

A stepwise forward multivariate logistic regression model was used to evaluate the risk factors for NOAF. As shown in Table 2, advanced age (odds ratio (OR):1.11, 95% CI 1.00–1.22, *p* = 0.045), increased plasma Gal-3 (OR 1.28, 95% CI 1.04–1.58, *p* = 0.018), and LAD (OR 1.27, 95% CI 1.05–1.54, *p* = 0.014) were independently associated with post-AMI NOAF, even after adjustment for other potential confounding risk factors.

**Table 2:** Logistic regression analysis of risk factors for post-MI NOAF

Variable	Univariate		Multivariate	
	OR (95% CI)	*p* value	OR (95% CI)	*p* value
Age	1.10 (1.04–1.17)	0.001	1.11 (1.00–1.22)	0.045
Gal-3	1.21 (1.09–1.34)	< 0.001	1.28 (1.04–1.58)	0.018
LDL-C	0.55 (0.31–0.95)	0.031	0.67 (0.27–1.59)	0.353
e-GFR	0.97 (0.95–0.99)	0.002	1.01 (0.98–1.05)	0.535
CRP	1.03 (1.01–1.04)	< 0.001	1.02 (0.99–1.04)	0.176
LVEF	0.92 (0.88–0.96)	< 0.001	0.96 (0.88–1.04)	0.275
LAD	1.59 (1.32–1.93)	< 0.001	1.27 (1.05–1.54)	0.014
BNP	1.00 (1.00–1.00)	< 0.001	1.00 (1.00–1.00)	0.086
Killip III-IV	5.4 (1.50–19.43)	0.010	0.15 (0.01–1.65)	0.120

OR, Odds ratio; CI, confidence interval; Gal-3, Galectin-3; LDL-C, low-density lipoprotein cholesterol; e-GFR, estimated glomerular filtration rate; CRP, C-reactive protein; LVEF, left ventricle ejection fraction; LAD, left atrial diameter

## Discussion

This study compared the baseline plasma Gal-3 concentration between AMI patients with and without post-AMI NOAF during the index hospitalization. The main findings were as follows: (1) baseline plasma Gal-3 concentration was significantly increased in patients with AMI with NOAF; (2) high plasma Gal-3 concentration and increased LAD were independent predictors of NOAF, even after adjustment for other confounders.

AF often coexists with coronary artery disease, and post-MI NOAF often occurs during the index hospitalization. NOAF is significantly associated with poor clinical outcomes. In the present study, 8.3% of all patients developed NOAF during hospitalization, which was consistent with previous findings [2].

The risk factors associated with post-MI NOAF have been under intensive focus in recent years. Lau et al. [13] demonstrated that advanced age, hypertension, HF, low e-GFR, high Killip level, and elevated C-reactive protein levels were independent risk factors for post-MI NOAF. Additionally, LV dysfunction and LA enlargement are significantly associated with post-MI NOAF [2, 13, 14]. In this study, we found that advanced age, a higher level of plasma Gal-3 concentration, and large LAD were independent predictors of post-MI NOAF during hospitalization.

In this study, we observed poor kidney function and a better lipid profile in patients who developed NOAF, which was consistent with the published literature. These phenomena may be attributed to the following: (1) Chronic kidney disease (CKD) has been reported to be significantly associated with the occurrence of NOAF after AMI [15]. Patients with CKD were older and often presented several aging diseases, which were significant risk factor for the occurrence of AF; (2) Cholesterol plays a key role in stabilizing the cell membrane, which directly determines the localization of ion channels, including the K + and Ca2 + subunits [16]. Low LDL levels may contribute to the incidence of NOAF by activating ion channels dysfunction, such as the K + and Ca2 + subunits, during AMI [17, 18].

In this study, β-blockers and ACEI/ARB were less used in those who developed NOAF than in those who did not, consistent with previous results [19, 20]. The underlying mechanisms were as follows: Cardiac remodeling has been confirmed to play a key role in the development of AF. Patients with a high degree of cardiac remodeling were at a significantly high risk of pre-stage of AF. AMI plays a key role in triggering the incidence of AF. That is why patients without β-blockers and ACEI/ARB were at a higher risk of AF.

The association between cardiac fibrosis and AF has been well established [21]. Gal-3 is a novel inflammatory biomarker secreted by activated macrophages and fibroblasts and plays a key role in the regulation of tissue fibrosis, including cardiac fibrosis. Due to the close correlation between inflammation and subsequent cardiac fibrosis and AF [22, 23], the association between Gal-3 and AF has been investigated in recent years. Gurses et al. [24] demonstrated that patients with AF had a significantly increased Gal-3 level than controls with sinus rhythm. A recent meta-analysis indicated that persistent AF patients had a significantly higher level of circulating Gal-3 concentration than paroxysmal AF patients [25]. Oluwaseun E et al. [9] conducted a large prospective population study and showed that a high level of plasma Gal-3 concentration (90^th^ percentile, 19.5 ng/ml) was significantly associated with an increased risk of incident AF. Accumulating evidence revealed that an increased level of Gal-3 was also significantly associated with AF recurrence after catheter ablation therapy [26, 27]. An experimental animal study found that after blocking Gal-3 function, structural remodeling of the atrial heart, characterized by excessive collagen deposition resulting from interstitial cardiac fibrosis induced by TGF-β/Smad3 signaling pathway, was significantly mitigated [8]. These phenomena were considered significant with an increased risk of AF.

As mentioned above, NOAF is significantly associated with a poor prognosis for patients with AMI, and Gal-3 is significantly associated with AF. Nonetheless, data on the association between post-MI NOAF and Gal-3 are limited. A recent case–control study revealed that AMI patients with pre-existing AF had a significantly higher Gal-3 level than those without AF [10]. Pavlovi et al. [28] demonstrated that plasma Gal-3 concentration was significantly higher in NSTEMI patients with preexisting AF than in those without preexisting AF, but Gal-3 was not significantly associated with composite outcomes. In addition, increased Gal-3 concentration was significantly associated with cardiac fibrosis and remodeling after MI [11]. To the best knowledge, this is the first study to evaluate the predictive value of Gal-3 for post-MI NOAF. The current results showed that AMI patients with NOAF had a significantly increased plasma Gal-3 concentration than those without NOAF. Multivariate logistic regression analysis indicated that a high concentration of Gal-3 in the plasma (optimal cut-off: 20.08 ng/ml) was an independent predictor of NOAF. Hernández-Romero et al. [29] reported that elevated plasma Gal-3 concentration (optimal cut-off point: 13.65 ng/ml) was independently associated with postoperative AF in patients undergoing elective cardiac surgery for cardiopulmonary bypass. The discrepancy between the two studies may be due to the different subject selection criteria; All subjects in our study were patients with AMI, which might influence the level of Gal-3. Additionally, in this study, we also observed a significantly higher level of Gal-3 in patients with STEMI than those with NSTEMI. Previous studies reported that the level of Gal-3 was higher in patients with unstable angina pectoris than in those with stable angina pectoris, and patients with a higher level of Gal-3 often had a tendency to have multivessel coronary artery disease [30, 31]. These evidences indicated that the Gal-3 level may be significantly associated with the degree of coronary stenosis.

In this study, we also evaluated the association between LAD and the incidence of post-MI NOAF and found that enlarged LAD was also an independent predictor of post-MI NOAF. For patients hospitalized with AMI, a transthoracic echocardiography test is an easy and timely method of cardiac evaluation. Enlarged LAD, a marker that reflects progressive dilatation and structural remodeling of the LA myocardium, acts as a key substrate in initiating and maintaining AF [32]. Increased plasma Gal-3 concentration promotes myocardial fibrosis by activating the TGF-β/Smad3 signaling pathway, leading to cardiac remodeling and LA dilation [33]. In addition to cardiac fibrosis, other factors, including hypertension and valvular disease, were also significant contributors to LA dilation. Advanced age was also independently associated with the incidence of NOAF after AMI during hospitalization, which was in line with previous findings [34, 35]. Increasing evidence has shown that aging and biomarkers of aging were significantly associated with AF [36]. The elderly often present many complications of aging-related diseases that might cause chronic structural and electrical remodeling of the atrium.

The mechanisms of post-MI AF could be partially explained by the fact that elevated Gal-3 concentration and enlarged LAD are indicators for preexisting atrial remodeling conditions, i.e., a pre-AF stage. Also, AMI might only be a trigger for the incidence of post-MI AF. Thus, the incidence of NOAF after AMI could be a surrogate for atrial remodeling. As shown in Table 3, NOAF patients did not receive more positive ACEI / ARB medications. Taken together, the present study hinted at the need for aggressive anti-remodeling medications such as angiotensin receptor-neprilysin inhibitors (ARNI) or sodium-glucose cotransporter-2 (SGLT2) inhibitors in patients with NOAF after AMI.

**Table 3:** In-hospital examination and treatment

Variables	Sinus rhythm (n = 199)	NOAF (n = 18)	*p*-value
*Laboratory test*			
CRP (mg/L)	5.0 (2.0, 18.2)	40.1 (1.6, 99.3)	0.231
LDL-c(mmol/L)	3.0 ± 1.0	2.5 ± 1.0	0.030
e-GFR (ml/min/1.73 m^2^)	97.2 ± 19.4	79.5 ± 31.1	< 0.001
Peak TnI(ng/ml)	36.0 (11.2, 108.6)	58.5 (31.0, 149.5)	0.293
Peak BNP (pg/ml)	188.0 (119.0, 331.0)	865.0 (398.0, 1977.0)	< 0.001
Gal-3(ng/ml)	16.6 ± 6.0	22.3 ± 5.1	< 0.001
*In-hospital medication*			
β-blockers	137 (68.8%)	7 (38.8%)	0.010
ACEI/ARB	79 (39.7%)	2 (11.1%)	0.016
*Echocardiography*			
LVEF (%)	59.2 ± 10.9	47.7 ± 11.9	< 0.001
LAD (mm)	35.3 ± 3.8	39.2 ± 3.9	0.004
LVESD (mm)	32.2 ± 5.6	36.4 ± 6.1	0.003
LVEDD (mm)	47.8 ± 4.2	50.7 ± 4.4	0.006

NOAF, new-onset atrial fibrillation; CRP, C-reactive protein; LDL-C, low-density lipoprotein cholesterol; e-GFR, estimated glomerular filtration rate; TnI, troponin I; Gal-3, Galectin-3; ACEI/ARB, angiotensin-converting enzyme inhibitors/ angiotensin receptor blocker; LVEF, left ventricle ejection fraction; LAD, left atrial diameter; LVESD, left ventricular end-systolic diameter; LVEDD, left ventricular end-diastolic diameter

## Limitations

Nevertheless, the present study had several limitations. First, the scale of the study population was small, and this was a single-center retrospective study; thus, a multip center perspective is essential with a large sample. Second, although we observed an association between plasma Gal-3 concentration and post-MI NOAF, this study could not establish a causal effect correlation. Third, no direct evidence of cardiac fibrosis was noted, but the LAD parameter was evaluated, which indirectly reflects cardiac remodeling. Finally, plasma Gal-3 concentration was not equal to the Gal-3 content of cardiac tissue Gal-3 content. However, the plasma concentration of Gal-3 was easily obtained and has been used widely for clinical research.

## Conclusions

Plasma Gal-3 concentration is an independent predictor of NOAF in patients with AMI during hospitalization. Thus, it could be a potential biomarker for NOAF risk stratification in AMI patients during hospitalization.

## Data Availability

The datasets generated and/or analyzed during the current study are not publicly available due to the restrictions of human genetics data policy of Beijing Chaoyang Hospital Ethics Committee, but are available from the corresponding author on reasonable request.

## Abbreviations

NOAF: New-onset atrial fibrillation
AMI: Acute myocardial infarction
Gal-3: Galectin-3
BNP: Brain natriuretic peptide
LDL-C: Low-density lipoprotein cholesterol
e-GFR: Estimated glomerular filtration rate
LVEF: Left ventricular eject fraction
LAD: Left atrial diameter
ROC: Receiver operating characteristic
AUC: Area under the curve
HF: Heart failure
PCI: Percutaneous coronary intervention
STEMI: ST-segment elevation myocardial infarction
NSTEMI: Non-ST-segment elevation myocardial infarction
